# Practical Notes on Diagnosis and Treatment

**Published:** 1907-12-21

**Authors:** 


### Practical Notes on Diagnosis and Treatment.

**Rheumatoid Arthritis.**

You will do well to begin treatment in this disease by the local application of heat and by massage. But very often you cannot do this, as people have to go about their business. Then, I believe, the best thing is plenty of good food and iodide of iron.—*Dr. Hale White.*

**Chicken-pox in the Adult.**

In chicken-pox in early life there is as a rule no severe febrile onset, but in the case of an adult the disease occasionally comes on with high temperatures, severe headache, and even pain in the back, and rigors are sometimes met with.—*Dr. Foord Caiger.*

**Mammary Cysts.**

A good way of treating a single cyst in the breast is to inject two or three drops of pure carbolic acid; even if fresh cysts form they can generally be got rid of in the same way. When, however, there are many cysts, it is commonly best to remove the affected part, or even the whole of the breast.

**Distension of the Bowel in Intussusception.**

I should advise you not to make use of distension at all, but to open the abdomen as soon as a diagnosis of intussusception is made. If you do, however, decide to try distension, use air, and try it only in very early cases, certainly not later than twenty-four hours from the onset of the symptoms.—*Mr. F. J. Steward.*

**Tonsillitis and Diphtheria.**

In follicular tonsillitis the exudate is, for the most part, only in spots; it does not often coalesce into a definite pellicle, and even if it does, it only rarely extends beyond the tonsil. I believe it sometimes encroaches on the back of the pharynx, but never, as far as I know, extends to the palate.—*Dr. Foord Caiger.*

**Enteric Fever.**

The enteric eruption is present in about 70 per cent. of the cases, and nearly all the remaining ones are children. During the early stage bronchial catarrh is frequent and is a very important sign of the disease; this is not sufficiently emphasised in text-books. Again, some degree of deafness is extremely common during the first week of enteric.—*Dr. Foord Caiger.*

**Asthma.**

In asthma and other forms of spasmodic dyspnœa paraldehyde is often very useful. It is best, in the case of adults, to give a dose of sixty minims at bed-time. The disagreeable flavour may be concealed by tincture of orange peel and cinnamon water.

**Neuralgia.**

In supra-orbital neuralgia large doses of quinine should be ordered. Another useful prescription in cases of neuralgia of the fifth nerve is five grains of butyl-chloral-hydrate with $\frac{1}{200}$ grain gelsemine.—*Dr. Murrell.*

**Hiccough.**

Two minims of a one per cent. solution of nitro-glycerine, with a drachm of spirit of chloroform, given in an ounce of water, repeated for three or four doses. This has proved successful treatment in cases of obstinate hiccough.

**Thyroid Extract.**

Amongst the conditions for which thyroid extract has proved useful are threatened abortion with hæmorrhage and subinvolution of the uterus. It has also some value as a galactagogue, and its use has been followed by shrinkage of uterine fibroids.

**Rheumatoid Arthritis and Gout.**

I lay stress on the fact of exostoses in true gout, and on that which is even more noteworthy—the occurrence of true ankylosis or bony synostosis in this disease, a condition which we do not find in rheumatoid arthritis.—*Sir Dyce Duckworth.*

**Chronic Diarrhœa.**

In some cases of chronic diarrhœa, where you fail to get any good at all from astringents, you may find that diarrhœa may be arrested by the use of a large belt to the abdomen, and more especially by a belt made of chamois leather.—*Sir Lauder Brunton.*

**Psoriasis.**

Salicin is the drug I use most largely in the treatment of psoriasis. The more acute and widespread the case the more likely are salicin and its congeners to be successful in the treatment of it. Begin with 15 grains three times daily directly after meals, and increase to 20 grains, or even more.—*Dr. Radcliffe Crocher.*

